# Integrative Computational and Experimental Approaches to Establish a Post-Myocardial Infarction Knowledge Map

**DOI:** 10.1371/journal.pcbi.1003472

**Published:** 2014-03-20

**Authors:** Nguyen T. Nguyen, Xiaolin Zhang, Cathy Wu, Richard A. Lange, Robert J. Chilton, Merry L. Lindsey, Yu-Fang Jin

**Affiliations:** 1 Department of Electrical and Computer Engineering, University of Texas at San Antonio, San Antonio, Texas, United States of America; 2 San Antonio Cardiovascular Proteomics Center, University of Texas Health Science Center at San Antonio, San Antonio, Texas, United States of America; 3 Center for Bioinformatics and Computational Biology and Protein Information Resource, University of Delaware, Newark, Delaware, United States of America; 4 Department of Medicine, University of Texas Health Science Center at San Antonio, San Antonio, Texas, United States of America; 5 Mississippi Center for Heart Research, University of Mississippi Medical Center, Jackson, Mississippi, United States of America; 6 Research Service, G.V. (Sonny) Montgomery Veterans Affairs Medical Center, Jackson, Mississippi, United States of America; The Centre for Research and Technology, Hellas, Greece

## Abstract

Vast research efforts have been devoted to providing clinical diagnostic markers of myocardial infarction (MI), leading to over one million abstracts associated with “MI” and “Cardiovascular Diseases” in PubMed. Accumulation of the research results imposed a challenge to integrate and interpret these results. To address this problem and better understand how the left ventricle (LV) remodels post-MI at both the molecular and cellular levels, we propose here an integrative framework that couples computational methods and experimental data. We selected an initial set of MI-related proteins from published human studies and constructed an MI-specific protein-protein-interaction network (MIPIN). Structural and functional analysis of the MIPIN showed that the post-MI LV exhibited increased representation of proteins involved in transcriptional activity, inflammatory response, and extracellular matrix (ECM) remodeling. Known plasma or serum expression changes of the MIPIN proteins in patients with MI were acquired by data mining of the PubMed and UniProt knowledgebase, and served as a training set to predict unlabeled MIPIN protein changes post-MI. The predictions were validated with published results in PubMed, suggesting prognosticative capability of the MIPIN. Further, we established the first knowledge map related to the post-MI response, providing a major step towards enhancing our understanding of molecular interactions specific to MI and linking the molecular interaction, cellular responses, and biological processes to quantify LV remodeling.

## Author Summary

Heart attack, known medically as myocardial infarction, often occurs as a result of partial shortage of blood supply to a portion of the heart, leading to the death of heart muscle cells. Following myocardial infarction, complications might arise, including arrhythmia, myocardial rupture, left ventricular dysfunction, and heart failure. Although myocardial infarction can be quickly diagnosed using a various number of tests, including blood tests and electrocardiography, there have been no available prognostic tests to predict the long-term outcome in response to myocardial infarction. Here, we present a framework to analyze how the left ventricle responds to myocardial infarction by combining protein interactome and experimental results retrieved from published human studies. The framework organized current understanding of molecular interactions specific to myocardial infarction, cellular responses, and biological processes to quantify left ventricular remodeling process. Specifically, our knowledge map showed that transcriptional activity, inflammatory response, and extracellular matrix remodeling are the main functional themes post myocardial infarction. In addition, text analytics of relevant abstracts revealed differentiated protein expressions in plasma or serum expressions from patients with myocardial infarction. Using this data, we predicted expression levels of other proteins following myocardial infarction.

## Introduction

Myocardial infarction (MI) is a prominent cause of mortality and morbidity worldwide [1]. MI is defined as the death of cardiac myocytes due to prolonged ischemia. As a result of myonecrosis, molecules from injured myocytes are discharged into the blood circulation, and the list of injury markers includes myoglobin, cardiac troponins T and I, creatine kinase-MB, and lactate dehydrogenase [2]. Molecular interactions within the myocardium activate a cascade of cellular responses, including a robust inflammatory response. The cellular responses within the LV are integrated by the extracellular matrix stimuli that bind to surface receptors. As such, the ECM coordinates the healing response to MI [3], [4], [5], [6], [7], [8].

Through the last 4 decades, there have been tremendous research efforts towards understanding the immediate myocyte response to ischemia, with the goal of identifying diagnostic indicators as well as targets to preserve myocyte viability. These have resulted in the implementation of several therapeutic strategies, including reperfusion and the use of angiotensin converting enzyme inhibitors [9], [10]. Currently, 30 day post-MI survival rates approach 90%, and the immediate prognosis is excellent for those patients who receive timely and effective treatment. The number of patients who will go on to develop congestive heart failure, in part as a consequence of this success, however, has increased. While much is known about the events that occur immediately before and after MI, much remains to be mechanistically elucidated regarding the effects of MI on long-term survival. A knowledge map that explores the regulatory relationship among ECM, cellular responses, and biological pathways post-MI is still lacking.

Over a million abstracts can be retrieved from PubMed using a keyword search for [“myocardial infarction” or “cardiovascular diseases”], and massive amounts of genomic and proteomic data and molecular profiles have been deposited in public databases [11], [12], [13]. High-throughput protein microarrays have provided efficient procedures to investigate and measure a vast number of protein-ligand interactions in a single experiment. Protein-protein interaction network (PPI) analysis using large-scale databases has been one of the most promising computational approaches to integrate experimental data at the molecular and cellular levels [14], [15], [16], [17]. Due to the growing availability of such large-scale datasets, PPIs have been applied to analyze numerous human diseases including lung cancer, breast cancer, and myocardial infarction [18], [19], [20].

The reported data which have largely been obtained with different experimental conditions, protocols, species, and research teams are embedded in the literature and distributed in disparate databases. The ability to integrate data from such heterogeneous resources will allow us to extract relevant information and identify knowledge gaps to direct future research efforts. To address these challenges, we report here an integrative computational approach including compiling a MI-specific PPI database through mining PubMed and UniProt to establish a knowledge map for LV remodeling post-MI [21], [22]. This MI-related knowledge map is the first major step towards enhancing our understanding of molecular interactions specific to MI and linking the molecular interaction, cellular responses, and biological pathways.

## Results

### The MI-specific protein-protein interaction network (MIPIN) is strongly connected

**Figure 1:**
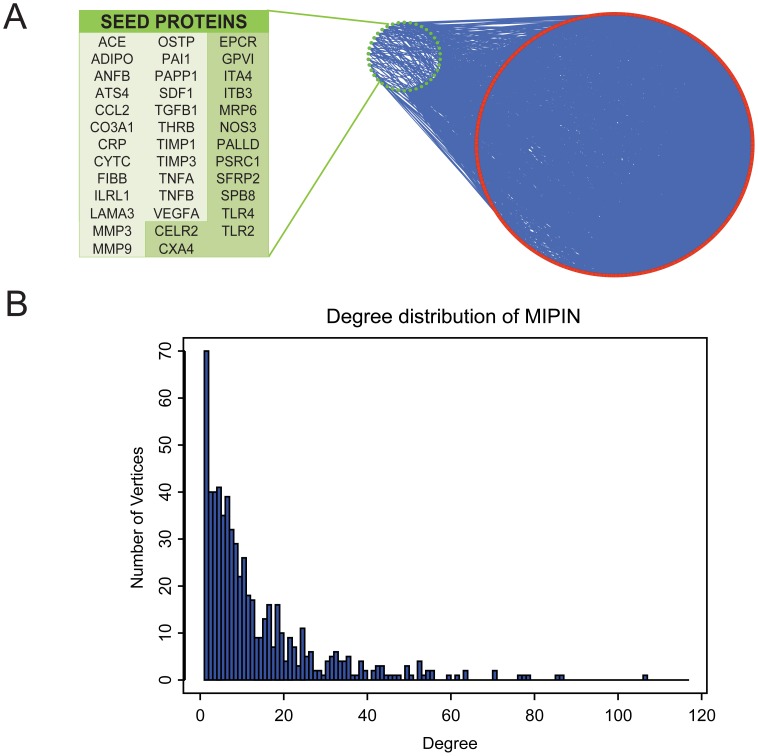
Structure of the MI-specific protein-protein interaction network (MIPIN). (**A**) Construction of the MIPIN from 38 seed proteins. Seed proteins are denoted as green circles while extended proteins (with interacting partners) are represented as red circles. Interactions are represented as blue edges. Seed proteins not localized in ECM were labeled with dark green background in the list. (**B**) Degree distribution of MIPIN. The histogram shows that the degree distribution of MIPIN followed a power law function, indicating that MIPIN is a scale-free network robust to disturbance. The degree ranged from 1 to 366, with polyubiquitin-C being an outlier with the highest degree and not included in the plot.

MI-related proteins were first obtained from the Online Mendelian Inheritance in Man (OMIM) database, PubMed Gene, and PubMed Protein databases by using “myocardial infarction” as the keyword and further refined by our cardiac clinicians (RAL and RJC) and cardiac biologist (MLL), producing a list of 38 seed proteins for humans [23]. With these seed proteins and their interacting proteins, we constructed a MI-specific PPI network with a total of 613 proteins (vertices) and an associated 4443 interactions (or edges) (Figure 1A). Detailed procedures to establish the MIPIN are provided in the *Methods*.

We observed that the MIPIN was strongly connected, in that there was always an edge between any two proteins in the MIPIN. Of the 613 proteins, 70 proteins had only 1 or 2 edges, 121 had 3 to 5 edges, and the rest had >5 edges. The degree distribution of MIPIN closely followed a power law distribution (Kolmogorov-Smirnoff test, *p-value* = 0.97, see *Methods* for details), where the degree of a vertex in a network was defined as the number of direct links incident upon that vertex (Figure 1B). The power law distribution indicated that the MIPIN was a scale-free network, which displayed robustness against disruptive failures of random vertices [24].

We performed two statistical tests to evaluate the specificity of the MIPIN. First, interactions were shuffled based on the Erdos-Renyi model, such that the 100,000 randomly generated networks each had 613 vertices and 4443 edges, which was the same number as the MIPIN [25]. Compared to the Erdos-Renyi model of random networks, the MIPIN had a lower average value of betweenness centrality while having higher average values of closeness centrality, clustering coefficient, and eccentricity (empirical *p-value*<0.001), indicating that proteins in the MIPIN were much more closely related to each other than would occur by random chance, and these proteins might have functional relevance.

**Figure 2:**
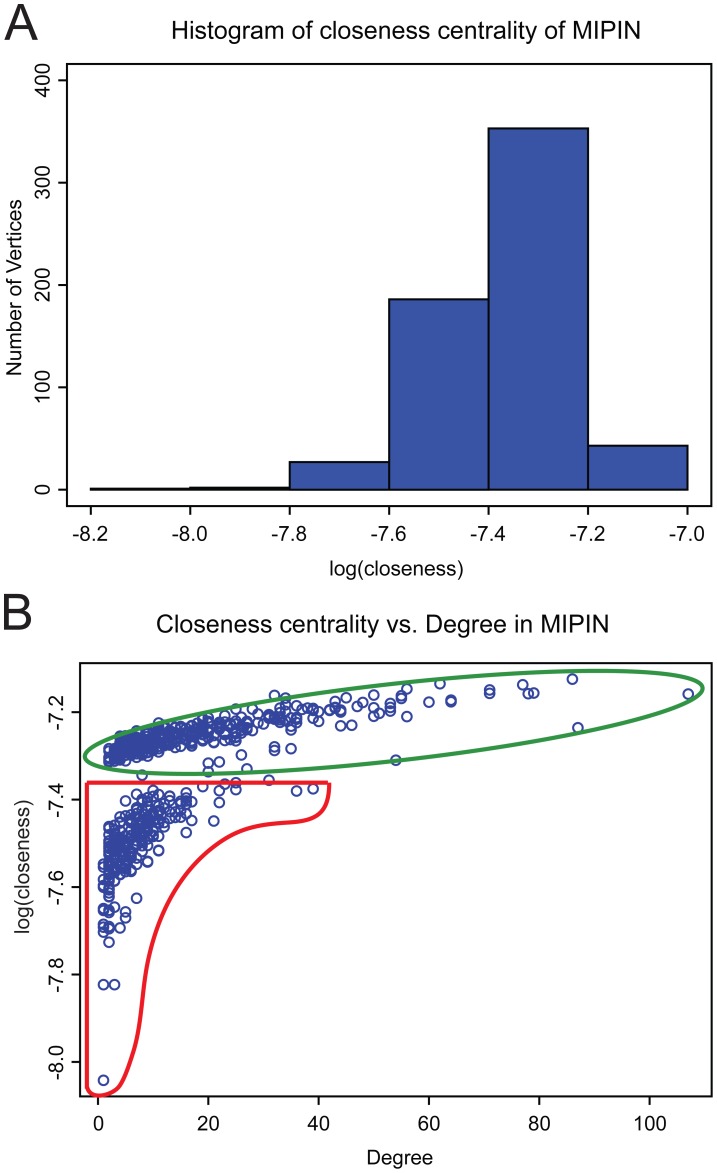
Specificity of the MIPIN. (**A**) Gaussian-like distribution of closeness centrality of MIPIN. (**B**) Closeness centrality vs. degree in MIPIN. Vertices having from 1 to 5 degrees displayed substantial differences in closeness centrality (red); on the other hand, as the degree of vertices increased, closeness centrality exhibited minor variation (green). These graphs demonstrate the clear differences between MIPIN and random networks (see also Figure S1 and S2).

In the second more stringent statistics test, we randomly picked the same number of seed proteins (*n* = 38) from 14969 human proteins and created 100,000 random networks in the same manner we constructed the MIPIN. Each random network had different number of vertices and edges. Compared to the randomly generated networks, the MIPIN had a higher mean value of closeness centrality and eccentricity (empirical *p-value*<0.05) and displayed a distinct distribution of closeness centrality (Figure 2A). We observed a Gaussian-like distribution for closeness centrality in the MIPIN, while closeness centrality distribution in the random networks resembled the Delta function with few vertices having very low value of closeness centrality, regardless of their number of vertices and edges (Figure S1). We also noticed that the vertices within a small range of degrees in the MIPIN had a larger variance of closeness centrality (Figure 2B), while the closeness centrality remained fairly constant with an increasing number of direct interactions in the random networks (Figure S2). Figure 2B shows that vertices in the first group [26] displayed substantial differences in closeness centrality with small changes of degree (natural logarithm of closeness centrality of the red group had a variance of 8.25×10^−3^). On the other hand, as the degree of a vertex increased, the closeness centrality exhibited minor variation (natural logarithm of closeness centrality of the red group had a variance of 1.37×10^−3^).

The overall structure of the MIPIN demonstrated that it was a strongly-connected and scale-free network, indicating that we captured a solid network of protein interactions from the human PPI that was highly specific. Further statistical tests allowed us to evaluate the significance of several MIPIN network properties, including betweenness centrality, closeness centrality, clustering coefficient, and eccentricity. The larger mean values of closeness centrality and eccentricity in MIPIN indicated that the randomly generated networks had more orphan sub-networks in contrast to the single strongly-connected MI network, suggesting proteins in MIPIN were significantly more closely related to each other and have more specific function than would occur by random chance.

### Proteins in the MIPIN are localized primarily in the extracellular matrix regions and plasma membrane

The localization of MIPIN proteins was determined using Gene Ontology (GO) enrichment analysis by DAVID [27], [28]. GO is a controlled vocabulary of terms that characterizes gene products in terms of their cellular components, biological processes, and molecular functions in a hierarchical structure from the most general to more specialized terms. The cellular components ontology describes locations at the levels of subcellular structures and macromolecular complexes. We focused on classification by cellular components to provide suggestions on the underlying physiological protein functions.

**Figure 3:**
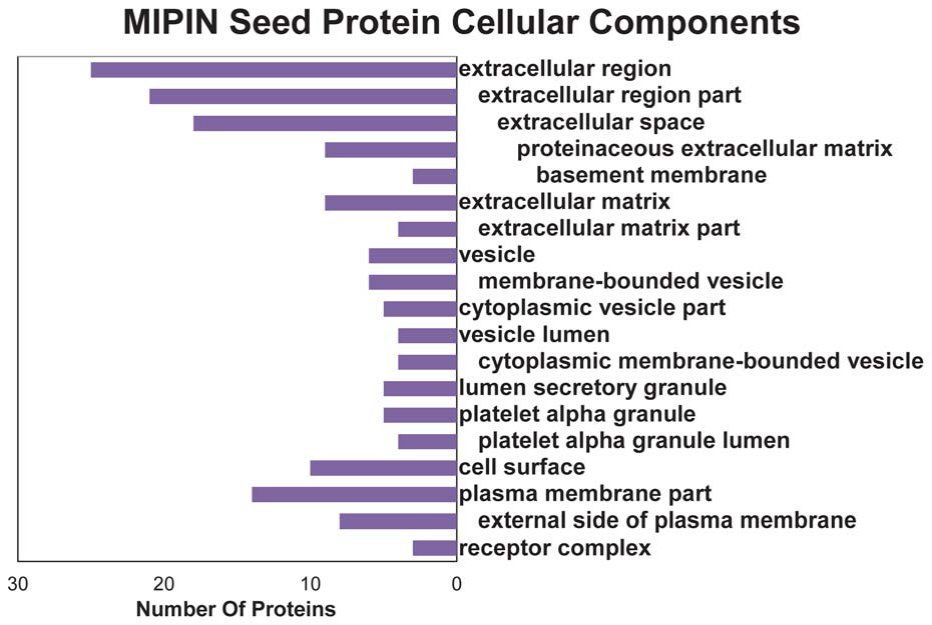
Localization of 38 MIPIN seed proteins. Seed proteins are localized in 19 locations, more than half of which are in the extracellular matrix (ECM) region. The indentation represents the cellular component hierarchy from the most general to more specialized terms. A horizontal blue line is used to separate ECM from cellular components.

More than 65% of the seed proteins were localized in the extracellular region, including vascular endothelial growth factor (VEGF), transforming growth factor beta-1 (TGFβ1), and tissue inhibitor of metalloproteinase-1 (TIMP1) (Figure 3). VEGF, TGFβ, and TIMP1 were also localized to platelet alpha-granules that have been known to play an important role in thrombosis, hemostasis, inflammation, atherosclerosis, wound healing, and angiogenesis [29]. In addition, VEGF, TGFβ, and TIMP1 were localized to the ECM, cell surface, and cytoplasmic membrane-bounded vesicle lumens in many cell types, suggesting active roles in multiple pathologies. A list of GO cellular components of the seed proteins were shown in Table S1.

**Figure 4:**
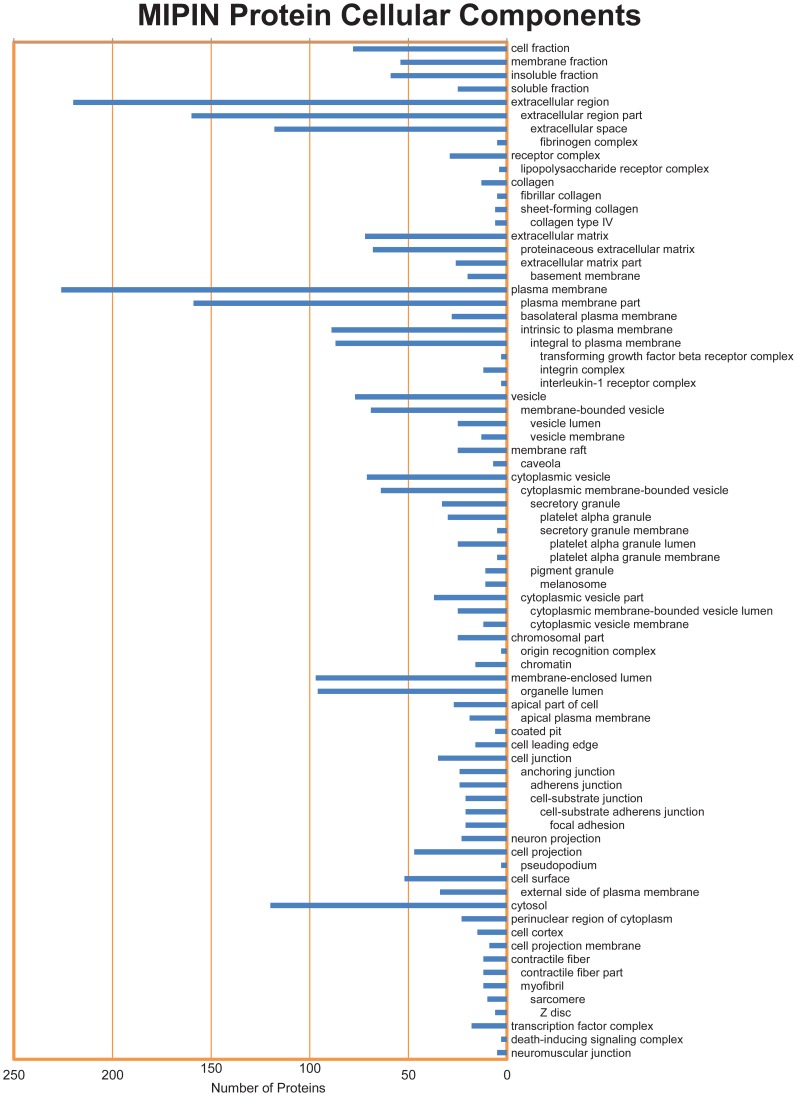
Localization of 613 MIPIN proteins. The complete set of MIPIN proteins (including interacting partners of seed proteins) are shown to be residing in 76 locations, with plasma membrane, extracellular region, and cytosol being the most preferred sites. The indentation represents the structural hierarchy of cellular component terms.

The inclusion of interacting partners of seed proteins in the MIPIN allows us to explore additional potential biomarkers for MI response. These proteins added 57 cellular components to the initial 19 locations (Figure 4). In addition to the extracellular region, the plasma membrane and cytosol were two preferred sites for most of the proteins in the MIPIN. We also identified a number of macromolecular complexes, including the TGFβ receptor complex, interleukin-1 (IL1) receptor complex, death-inducing signaling complex, origin recognition complex, lipopolysaccharide receptor complex, fibrinogen complex, integrin complex, and transcription factor complex. These complexes strongly suggest the presence of an inflammatory response. The signaling pathway of the lipopolysaccharide receptor complex has been linked to activation and deactivation of macrophages by lipopolysaccharide, a major cell responding to inflammation [30]. Activated macrophages secrete many different inflammatory cytokines, including IL1 and TGFβ. IL1 receptor complex and TGFβ receptor complex are essential factors in the inflammatory response post-MI [31], [32].

### Transcription activity, ECM remodeling, and inflammatory response are main functional themes of the MIPIN

We found 993 enriched GO biological process terms associated with MIPIN using DAVID. To glean functional insight from the large number of enriched GO biological process terms, we adapted a method from Louie et al. to extract the most meaningful biological processes, in terms of specificity [33]. In the GO structural hierarchy, the biological processes can be traversed from the root/parent node (GO:0008150:“biological process”) to narrower and more specific definitions in the child nodes, such as from the parent node “regulation of blood coagulation” to its child terms: “positive regulation of blood coagulation” and “negative regulation of blood coagulation”.

The function specificity for the GO terms was evaluated based on four measures: number of ancestor terms, offspring score, proportion of terms, and information content. Higher values of these measures indicate higher specificity. A broader, more general term has less number of ancestor terms and more offspring when compared to a narrower, more specific definition. The broadest term “biological process” had no ancestors, since it is the root node in the biological process branch, as the parent of all other GO biological process terms. The offspring score for a GO term was calculated based on the number of offspring for a node such that a higher score represents more specific function. GO proportion described the ratio between numbers of ancestor and offspring terms, with 0 indicating non-specific and 1 indicating the highest specificity. In addition, we considered the probability of observing a GO term because more specific terms annotate less number of genes, and thus were less likely to be found enriched in a dataset. Information content (IC) was a normalized score of this probability such that the root node has an IC of 0, and more specific terms have higher IC.

**Figure 5:**
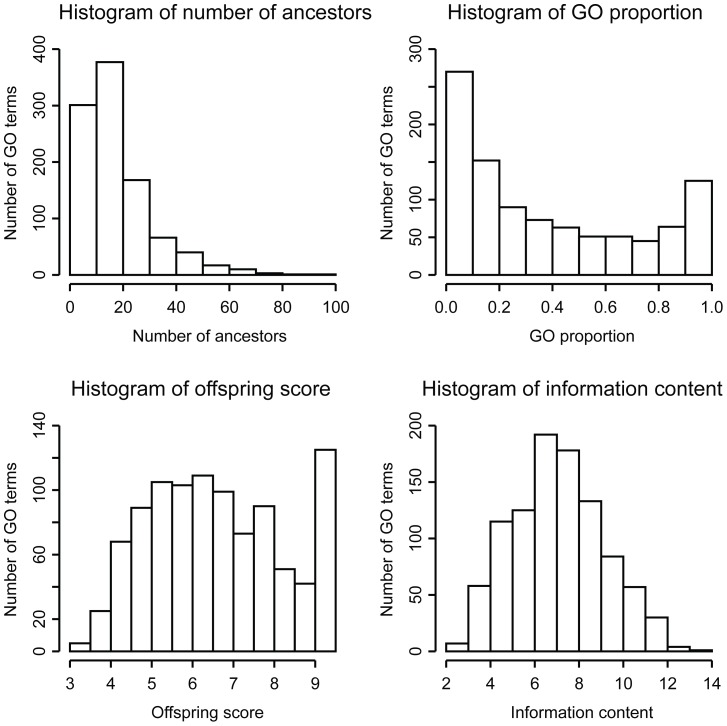
Specificity of GO biological process terms. (**Top left**) Histogram of number of ancestors. (**Bottom left**) Histogram of number of offspring. (**Top right**) Histogram of GO proportion. (**Bottom right**) Histogram of information content.

We obtained very different distributions of the 993 biological process GO terms for each of these measures (Figure 5). The number of ancestors followed a power-law distribution while information content followed a Gaussian-like distribution. These four evaluations illustrated that only a small number of 993 GO terms were specific. Among the most specific GO terms with regards to the number of ancestors, the top 20 terms were related to kinase and transcriptional activities, suggesting the significant signaling in the MIPIN (Table S2). We obtained 80 enriched GO terms that had only one offspring in the GO dataset while the offspring of the 80 GO terms were not enriched Table S3). These 80 GO terms were the most specific biological processes we could identified for MIPIN. These terms also emphasized the role of kinase signaling, cell apoptosis/necrosis, migration, differentiation, cell-matrix adhesion, ECM remodeling, and inflammatory response. Top 20 GO proportion evaluation resulted in significance of kinase activity and inflammatory responses (Table S4). The top 20 biological processes with the highest IC score highlighted inflammatory and immune responses (Table S5). The top two terms in the IC list were “negative regulation of L-glutamate transport” (*p-value*<0.01) and “regulation of L-glutamate transport” (*p-value*<0.05). Currently, there are very few studies on the role of L-glutamate post-MI. Lofgren et al. found that L-glutamate provides cardioprotection in the same manner as classical ischemic preconditioning [34].

We listed the most significant GO biological process terms based on the four specificity measures and noticed that transcription activity, response to inflammation, and ECM remodeling accounted for the most significant processes (*p-value*<0.0001, Table 1). “Positive regulation of JUN kinase activity” (*p-value*<0.01) had the highest GO proportion as of 0.987, the most number of ancestors (81) and only one child term, and a relatively high IC score as of 7.96, therefore, we identified it as one of the most enriched GO terms in the MIPIN. “Positive regulation of interleukin-6 biosynthetic process” and “positive regulation of interleukin-12 biosynthetic process” (*p-value*<0.005) ranked among the top GO terms with highest number of ancestors, GO proportion and IC score. These two processes represent inflammatory response post-MI. Additionally, three other inflammatory functions “activation of plasma proteins involved in acute inflammatory response”, “connective tissue replacement involved in inflammatory response wound healing” and “wound healing involved in inflammatory response” (*p-value*<0.0001) were ranked high in the top 20 IC list, further confirming the importance of inflammatory response post-MI. These pathways are also important for wound healing. Together with collagen fibril organization and cell-matrix adhesion GO terms, we identified ECM remodeling as another key component post-MI.

**Table 1:** The most significant GO biology process terms based on four specificity measures (number of ancestors, offspring score, GO proportion, and information content).

GO ID	Term	Number of Ancestors	Offspring score	GO proportion	Information Content
**GO:0043507**	positive regulation of JUN kinase activity	81	9.389	0.987	7.96
**GO:0051092**	positive regulation of NF-kappaB transcription factor activity	41	9.389	0.976	7.099
**GO:0032760**	positive regulation of tumor necrosis factor production	14	9.389	0.933	8.796
**GO:0048661**	positive regulation of smooth muscle cell proliferation	14	9.389	0.933	8.483
**GO:0001954**	positive regulation of cell-matrix adhesion	17	9.389	0.944	9.256
**GO:0030199**	collagen fibril organization	9	9.389	0.9	8.631
**GO:0002541**	activation of plasma proteins involved in acute inflammatory response	15	9.389	0.938	12.256
**GO:0045410**	positive regulation of interleukin-6 biosynthetic process	43	9.389	0.977	11.034
**GO:0045084**	positive regulation of interleukin-12 biosynthetic process	43	9.389	0.977	11.034
**GO:0002248**	connective tissue replacement involved in inflammatory response wound healing	11	9.389	0.917	12.256
**GO:0002246**	wound healing involved in inflammatory response	8	8.982	0.8	11.519

### Integrating experimental results to predict protein expressions post-MI with the MIPIN

Based on GO biological process information and MIPIN structure, we predicted protein expression levels in the MIPIN and validated with published results obtained from MI patient data. We automatically text-mined plasma and serum protein expression levels in post-MI patients reported in articles published between Jan 1, 2005 and May 31, 2013. We chose plasma and serum measurements here for an easier clinical study in the future. Abstracts studying association of MI with diabetes, or coronary artery diseases without MI, or protein concentrations being measured after percutaneous coronary intervention post-MI, were not considered. R and Java programs were written to perform XML parsing and text mining on relevant PubMed abstracts (see *Methods*). From a total of 4326 abstracts, we obtained 21 highly confident up-regulated proteins, and 1 down-regulated protein (Adiponectin), each with expression results confirmed by at least 2 citations (Table S6).

We used a semi-supervised learning method to predict expression changes in other proteins in the network. With the available expression levels on 22 “labeled” proteins as the training set, we predicted 14 up-regulated proteins (Table 2). To validate the computational predictions, we examined reported literature from 1990 till current and found that 11 of the 14 predicted proteins have supporting experimental evidence. Stromelysin-1 (matrix metalloproteinase-3 [MMP3]), neutrophil elastase (also named as Human leukocyte elastase, HLE), thrombospondin-1 (TSP1), and fibronectin [35] increased in plasma from patients post-MI [36], [37], [38], [39], [40]. In mouse models of MI, CD44 increased in LV by 6 hours, C-C motif chemokine 7 (CCL7) increased in ischemic myocardium after 24 hours, ELAV-like protein 1 [41] increased as well as matrilysin (MMP7) [42], [43], [44], [45]. Inhibition of collagen XVIII (COIA1) was found to impair LV remodeling and heart failure in rat MI model [46]. While there was no available expression data on complement factor H (CFAH) and matrix metalloproteinase-17 (MMP17) in plasma from patients post-MI, the CFAH polymorphism Y402H has been inversely associated with the risk of coronary heart disease (CHD) among women but not men, and MMP17 was found to be overexpressed in atherosclerotic vessels [47], [48]. We did not find any information regarding TIMP3, TNF-receptor associated factor 6 (TRAF6), and brevican core protein (PGCB) in the setting of MI either for human or animal studies, although TIMP3 was down-regulated in patients with ischemic cardiomyopathy (ICM) and dilated cardiomyopathy (DCM) [49]. Further experimental measurements on these proteins are needed to validate our predictions post-MI.

**Table 2:** Predicted up-regulated proteins, based on expression levels of 22 labeled proteins and summaries of the validating results with details on species, tissue, and references.

Protein	Disease	Model	Tissue	Change	References
*CCL7*	MI	Mice	LV	Increased in ischemic myocardium 24 h post MI	[43]
*CD44*	MI	Mice	LV	Increases in LV post MI by 6 h and starts reducing by 24 h	[42]
*CFAH*	Coronary heart diseases	Human	Plasma	Inversely associated with the risk of coronary heart diseases among women, but not men	[48]
*COIA1*	MI	Mice	LV	-	[46]
*HUR*	MI	Mice	LV	Increased	[44]
*HLE*	MI	Human	Plasma	Increased and peaked around 40 h post-MI	[37], [38]
*FN*	MI	Human	Plasma	Increased from 12 h to 14 days post-MI	[40]
*MMP17*	Atherosclerosis	Human	Aortic wall	Overexpression	[47]
*MMP3*	MI	Human	Plasma	Slowly increased with time (0–12 h; 12–24 h; 24–48 h; 48–72 h; 72–96 h). After 48 h, MMP-3 levels were significantly higher (vs 0 h)	[39]
*MMP7*	MI	Mice	LV infarct and LV noninfarct	3-fold higher in remote and infarct regions at day 7 post-MI	[45]
*PGCB*	-	-	-	-	-
*TIMP3*	*Ischemic/Dilated Cardiomyopathy*	Human	LV	Reduced in Ischemic and Dilated Cardiomyopathy	[49]
*TRAF6*	-	-	-	-	-
*TSP1*	MI	Human	Plasma	Increased	[36]

**Figure 6:**
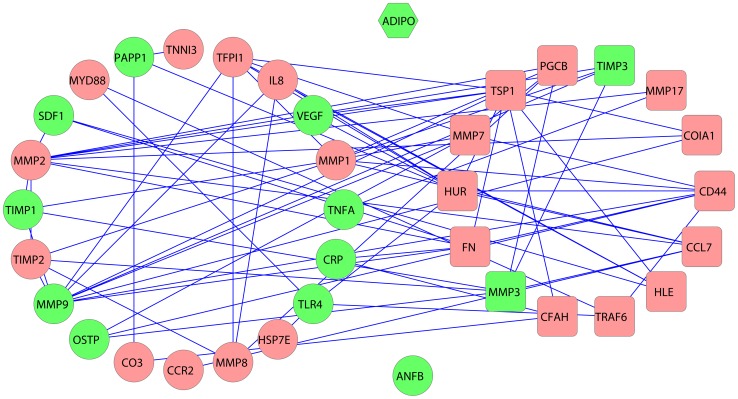
Interaction between labeled proteins with predicted proteins. Known down-regulated proteins are represented as hexagons. Known up-regulated proteins are represented as circles. Predicted up-regulated proteins are represented as rounded rectangles. Green nodes indicate seed proteins, and red nodes indicate extended interacting proteins.

The interactions among the 36 proteins were shown in Figure 6. All 14 predicted proteins and 22 labeled proteins are well connected, except two labeled proteins (ADIPO and ANFB). Since Adiponectin (ADIPO) was the only down-regulated protein post-MI, we did not have sufficient evidence to predict other down-regulated proteins. Also, we could not use natriuretic peptides B (ANFB, also named as BNP for gene name) to predict any proteins because none of its direct neighbors were connected to proteins with known quantifications, hence having low predictive confidence.

### Establishment of MI knowledge map

**Figure 7:**
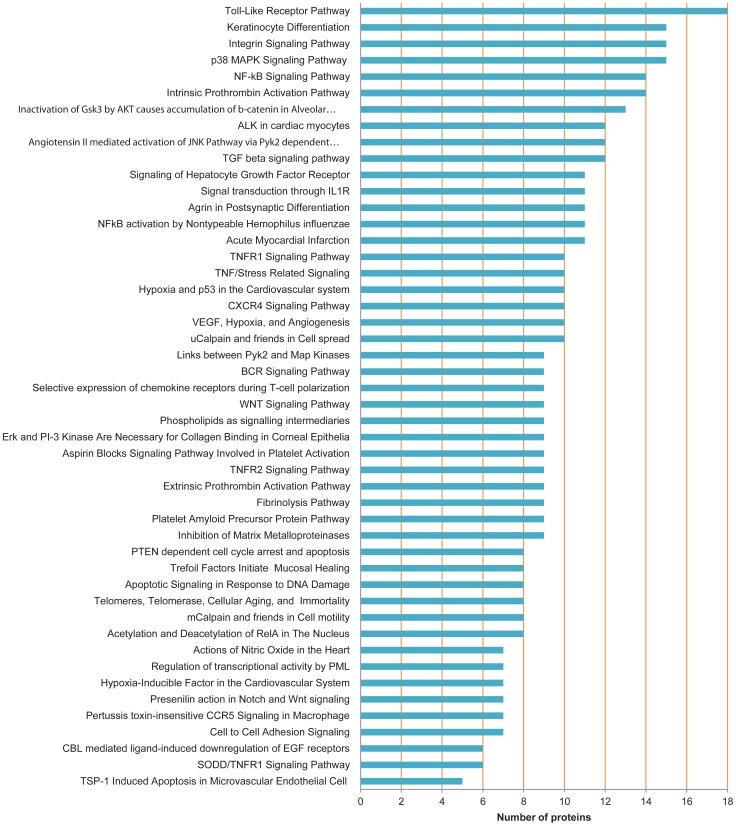
Enriched Biocarta pathways of MIPIN proteins. The number of proteins in each pathway is shown as the horizon coordinate.

Although the GO biological process revealed the overall underlying molecular functions, it could not capture the regulatory dynamics and dependencies required to completely describe a pathway. To have a better understanding of MI pathology, we examined the 613 proteins in the MIPIN and found 48 highly enriched pathways from Biocarta (http://biocarta.com/; Figure 7). These pathways covered broad categories, including adhesion, apoptosis, cell activation, cell cycle regulation, cell signaling, cytokines/chemokines, developmental biology, expression, hematopoiesis, and immunology.

**Figure 8:**
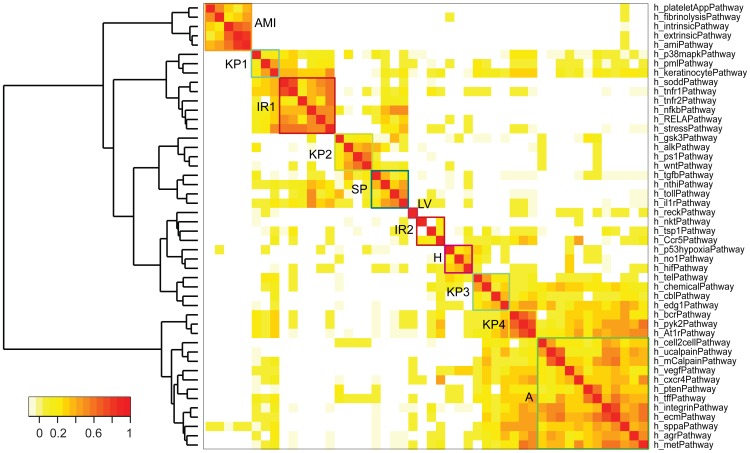
Heat map of Kappa similarity matrix for enriched Biocarta pathways. The graph visualizes the similarity of different pathways using Kappa statistics (see *Methods* for details). At the cutoff value of 2.5, we identified 10 clusters. Checking protein functions in these pathways, we grouped these clusters into 7 components, including **K**inase **P**athways (**KP1–4** in chartreuse), **A**ngiogenesis (**A** in green), **A**cute **MI** (**AMI** in orange), **I**nflammatory **R**esponses (**IR1–2** in red), **H**ypoxia (**H** in magenta), **LV** remodeling (**LV** in dark red), and other **S**ignaling **P**athways (**SP** in dark green). Colors for the clustering boxes are matched for Table 3, Table S8 and, Figure S3.

We clustered the 48 enriched Biocarta pathways with respect to their Kappa similarity matrix into 10 functional groups including 4 groups of Kinases Pathways, Angiogenesis, Hypoxia, Acute MI, 2 groups of Inflammatory Responses, LV Remodeling, and other Signaling Pathways (Figure 8).

Each row and column in Figure 8 represented an enriched Biocarta pathway for MIPIN. The sequence of pathways in rows and columns are the same. The row sequence of pathways was shown from the top to the bottom in Figure 8. Each cell in the figure represented the intersection between a row and a column and the color of a cell represented the similarity between two pathways. The color legend denoted the similarity between two pathways with the red representing high similarity and light color representing low similarity. The strongest similarity was the self-similarity and the color blocks with deepest red color were located on the diagonal of this symmetric figure.

It was shown that the acute MI group (block AMI) shared high similarity within the block and relative low similarity with only two pathways h_sppaPathway in block angionenesis (block A) and h_p53hypoxiaPathway in block hypoxia (block H). h_sppaPathway denoted “aspirin blocks signaling pathway involved in platelet activation” and h_p53hypoxiaPathway denoted the role of p53 and hypoxia in the cardiovascular system. Interestingly, by checking the color of the intersections of h_p53hypoxiaPathway and h_sppaPathway, the similarity between these two pathways were very low, suggesting no proteins in common in these two pathways and these two pathways could independently contribute to acute MI.

Kinases (KP) and signaling pathway (SP) blocks shared high similarity with more pathways in general since they transmitted spatial signals to trigger pathways related to cellular functions, which was illustrated by the appearance of light yellow boxes in the rows/columns representing KP and SP blocks. Specifically, kinases pathway blocks KP1, KP2, and signal transduction pathway SP were closely related to inflammatory response IR1. Kinases pathway block KP3 was closely related to hypoxia block H. Kinases pathways block KP4 was closely related to angiogenesis block. As an example, platelet activation (h_sppaPathway) was one of the pathways that shared similarity with the most number of pathways Figure 8. It shared higher similarity with kinases pathway block KP1 and low similarity with inflammatory response block IR1 (as shown in the 3^rd^ column from the right or 3^rd^ row from the bottom). Meanwhile, KP1 and IR1 shared high similarity, suggesting a cause-effect relationship from platelet activation, kinases pathway KP1 to inflammatory response IR1 cascade. Platelet activation pathway also shared high similarity with KP3, KP4, and angiogenesis (A) blocks, suggesting a possible regulation between platelet activation and angiogenesis.

Although there was no specific pathway named LV remodeling in Biocarta, we defined the Inhibition of Matrix Metalloproteinases pathway (h_reckPathway) as part of LV remodeling in our knowledge map since the pathway was closely related to ECM degradation. There are 9 proteins listed in pathway by Biocarta, including MMP-2, -9, TIMP-1, -2, -3, -4, reversion-inducing-cysteine-rich protein with kazal motifs (RECK), v-Ha-ras Harvey rat sarcoma viral oncogene homolog (RAS) and all of them were included in our MIPIN. This pathway did not show high similarity with any other pathways in Figure 8 though illustrating low similarity with h_pmlPathway in KP1 block, h_bcrPathway and h_pyk2Pathway in KP4 block, and 7 pathways in angiogenesis block, suggesting possible regulation among LV remodeling, inflammatory response, and angiogenesis.

To better understand Figure 8, pathways clustered in each functional group were listed in Table 3, and 160 proteins with specific regulatory relationship in each functional group were listed in Table S8. This forms the basic knowledge map for MI response that links proteins to specific pathways and functional groups. Combining functional information for all 613 potential MI related proteins extracted by MIPIN, including cellular components, biological processes, and specific pathways, we established the knowledge map for MI (Figure S3). Essentially, the knowledge map summarizes important spatial and temporal aspects of the static MIPIN; it describes the progression of MI and involvement of different proteins in three major phases: Development of MI (hypoxia and acute MI), response to MI (signaling pathway, kinases pathway, and inflammatory responses), and tissue remodeling (left ventricle remodeling and angiogenesis).

**Table 3:** Biological processes enriched with the clustered pathways using Kappa similarity matrix.

Biological processes enriched by clustered pathways	Enriched 48 Biocarta pathways
Acute MI (AMI)	h_plateletAppPathway, h_fibrinolysisPathway, h_intrinsicPathway, h_extrinsicPathway, h_amiPathway
Kinases Pathways (KP1)	h_p38mapkPathway, h_pmlPathway, h_keratinocytePathway
Inflammatory Response (IR1)	h_soddPathway, h_tnfr1Pathway, h_tnfr2Pathway, h_nfkbPathway, h_RELAPPathway, h_stressPathway
Kinases Pathways (KP2)	h_gsk3Pathway, h_alkPathway, h_ps1Pathway, h_wntPathway
Signaling Pathways (SP)	h_tgfbPathway, h_nthiPathway, h_tollPathway, h_il1rPathway
LV Remodeling (LV)	h_reckPathway
Inflammatory Response (IR2)	h_nktPathway, h_tsp1Pathway, h_Ccr5Pathway
Hypoxia (H)	h_p53hypoxiaPathway, h_no1Pathway, h_hifPathway
Kinases Pathways (KP3)	h_chemicalPathway, h_cblPathway, h_edge1Pathway, h_telPathway
Kinases Pathways (KP4)	h_bcrPathway, h_pyk2Pathway, h_At1rPathway
Angiogenesis (A)	h_cell2cellPathway, h_uCalpainPathway, h_mCalpainPathway, h_vegfPathway, h_cxcr4Pathway, h_ptenPathway, h_tffPathway, h_integrinPathway, h_ecmPathway, h_sppaPathway, h_agrPathway, h_metPathway

## Discussion

The goal of this study was to establish a framework to 1) automatically extract the information embedded in MI-related PubMed abstracts and reported data through a PPI network, 2) integrate the information into a knowledge map for MI response, and 3) cluster proteins in the knowledge map based on their functions. In this study, we started from the seed proteins for MI and PPI databases at molecular level, extended to cellular components of the proteins at cellular level, and further mapped the information to functional responses and specific pathways to illustrate a complete framework that integrates molecular, cellular, and functional analysis.

There are three major contributions of this study. First, we established a MI-specific PPI network and confirmed its specificity with two different statistical analyses. We predicted expression levels of 14 proteins in the MIPIN based on the up/down regulations of 22 proteins. The predicted protein expressions from computational analyses agreed well with reported experimental measurements. Second, we illustrated the importance of inflammatory and ECM remodeling responses in LV remodeling post-MI. Most proteins in the MIPIN were localized primarily in the extracellular regions and the plasma membrane. Additionally, transcription activity, ECM remodeling, and inflammatory response were the main functional themes of the MIPIN. In fact, almost half of the 22 highly confident proteins were inflammatory or extracellular proteins, demonstrating that these two phases are very crucial in determining the outcome of MI. Third, we established the first knowledge map for MI response based on the clustered pathways. This is the first knowledge map constructed by integrating our knowledge obtained from molecular, cellular, and functional factors via PPI, cellular components, biological processes and pathways. In addition, the knowledge map illustrated the temporal response from development of MI to tissue remodeling and the related proteins at each stage. The approach to establish the knowledge map for MI could also be applied to other diseases.

Our results illustrated that using the structural property of the PPI network is a promising technique to distinguish functional specific networks from random networks. However, individual structure property alone may not be sufficient to identify significant markers. Degree centrality provides independent evaluation of direct links of a vertex. Intuitively, a hub protein with higher degree may represent a significant marker. However, this cannot be confirmed with current clinical practice. For example, cardiac troponin I (cTnI) is a well-known biomarker for MI but cTnI only has a degree of 3 in our network [50]. Additionally, MMP9 and TIMP1 have been reported as key regulators of LV remodeling post-MI in a number of publications, while MMP9 had a degree of 36 and TIMP1 had 12, the average degree of MIPIN was 15 [51], [52]. Another structure property, betweenness, denotes how frequently a vertex or edge is used while walking through the network with shortest path. The combination of different structural properties might be a promising way to identify key markers. For example, a vertex with small degree and high betweenness denotes a protein that is frequently used to transmit information in the network, suggesting its significance as a bottle neck of the network or cross talk between biological processes. More accurate analysis of such evaluation scheme will be conducted in our future research.

Our results highlight the influence of the early inflammatory response initiated after tissue hypoxia. Following hypoxia, up-regulation of RAS, focal adhesion kinase 1 (FADK1), paxillin (PXN), and p53 simultaneously induce at least four major cellular activities, including cell proliferation, migration, apoptosis and necrosis. Proliferation of endothelial cells increases the production of nitric oxide (NO), which plays an important role in the later phase of LV remodeling and wound healing. Fibroblasts and myofibroblasts deposit a network of collagen at the infarct site, preparing for the formation of tissue granulation. Collectively, cell proliferation, migration, apoptosis and necrosis contribute to angiogenesis parallel to scar formation.

In summary, we report here the establishment of the first MI-specific PPI network that can be used as a foundation to interrogate the literature for candidate biomarkers of adverse remodeling post-MI.

## Methods

### Selection of seed proteins for MIPIN

In order to acquire a list of proteins related to MI, we initiated a keyword search for “myocardial infarction” in three different databases including OMIM, PubMed Gene and PubMed Protein, resulting in an initial pool of 658 genes from OMIM and PubMed Gene and 2319 protein sequences from PubMed Protein databases. Because the obtained genes were retrieved using both animal and clinical studies, all the genes and proteins retrieved from OMIM, PubMed Gene, and PubMed Protein databases were matched for human protein names in UniProt, yielding 709 proteins (Table S9). By evaluating the description of the genes obtained from OMIM, terms not related to MI response were revealed (e.g., stroke, arrhythmogenic, cardiomyopathy, and arterial calcification). These genes were removed from our list. We also removed proteins directly related to myocytes, since these proteins reflect more the pre-MI or acute MI instead of post-MI response. From this, we were left with 22 MI response related genes.

Searching PubMed Gene and Protein databases provides a candidate list of genes and protein sequences potentially associated with MI; however, this search strategy does not provide any description of the retrieved genes and proteins. We verified additional 16 seed proteins associated with MI using genome wide disease association databases, GENERIF and PubMed. This led to a total of 38 seed proteins including the major ones previously identified in our experiments, including collagen, MMP9, TIMP1, TNFα, TGFβ, and monocytes chemotactic protein-1 (MCP1). All seed proteins were associated with MI in at least 2 independent manuscripts, as shown in Table 4.

**Table 4:** The list of seed proteins with references to confirm the association of the proteins with MI.

Uniprot ID	Source	Evidence	Selected Publications
*ACE*	OMIM/PubMedGene/PubMedProtein	OMIM text/GENERIF/GENETIC_ASSOCIATION_DB_DISEASE	[64], [65], [66]
*ADIPO*	OMIM/PubMedGene	OMIM text/GENERIF/GENETIC_ASSOCIATION_DB_DISEASE	[67], [68], [69]
*ANFB*	PubMedGene	GENERIF	[70], [71], [72]
*ATS4*	PubMedGene/PubMedProtein	GENERIF	[73], [74], [75], [76]
*CCL2*	PubMedGene/PubMedProtein	GENERIF/GENETIC_ASSOCIATION_DB_DISEASE	[77], [78], [79]
*CELR2*	PubMedGene	GENERIF	[80], [81], [82]
*CO3A1*	OMIM	OMIM text	[83], [84], [85]
*CRP*	OMIM/PubMedGene/PubMedProtein	OMIM text/GENERIF/GENETIC_ASSOCIATION_DB_DISEASE	[71], [86], [87]
*CXA4*	OMIM/PubMedGene/PubMedProtein	OMIM text/GENERIF/GENETIC_ASSOCIATION_DB_DISEASE	[88], [89], [90]
*CYTC*	PubMedGene	GENETIC_ASSOCIATION_DB_DISEASE	[91], [92], [93]
*EPCR*	OMIM/PubMedGene/PubMedProtein	OMM text/GENERIF/GENETIC_ASSOCIATION_DB_DISEASE	[94], [95], [96]
*FIBB*	OMIM/PubMedGene	OMIM text/GENETIC_ASSOCIATION_DB_DISEASE	[97], [98], [99]
*GPVI*	PubMedGene	GENERIF/GENETIC_ASSOCIATION_DB_DISEASE	[100], [101], [102]
*ILRL1*	PubMedGene	GENERIF	[103], [104], [105]
*ITA4*	PubMedProtein	PubMed citations	[106], [107], [108]
*ITB3*	OMIM/PubMedGene	OMIM text/GENETIC_ASSOCIATION_DB_DISEASE	[109], [110], [111]
*LAMA3*	PubMedGene	GENERIF	[112], [113], [114]
*MMP3*	OMIM/PubMedGene	OMIM text/GENETIC_ASSOCIATION_DB_DISEASE	[39], [115], [116]
*MMP9*	OMIM/PubMedGene	OMIM text/GENETIC_ASSOCIATION_DB_DISEASE	[117], [118], [119]
*MRP6*	OMIM/PubMedGene	OMIM text/GENETIC_ASSOCIATION_DB_DISEASE	[41], [120], [121]
*NOS3*	PubMedGene	BIOCARTA/GENERIF/GENETIC_ASSOCIATION_DB_DISEASE	[97], [122], [123]
*OSTP*	PubMedGene	GENERIF	[124], [125], [126]
*PAI1*	OMIM/PubMedGene/PubMedProtein	OMIM text/GENERIF/GENETIC_ASSOCIATION_DB_DISEASE	[95], [127], [128], [129]
*PALLD*	OMIM/PubMedGene/PubMedProtein	OMIM text	[130], [131]
*PAPP1*	OMIM/PubMedGene	OMIM text/GENERIF	[132], [133], [134]
*PSRC1*	PubMedGene	GENERIF	[12], [80], [82]
*SDF1*	OMIM/PubMedGene	OMIM text/GENERIF/GENETIC_ASSOCIATION_DB_DISEASE	[135], [136], [137]
*SFRP2*	OMIM/PubMedGene	OMIM text	[138], [139], [140]
*SPB8*	PubMedProtein	PubMed citations	[141], [142]
*TGFB1*	PubMedGene	GENERIF/GENETIC_ASSOCIATION_DB_DISEASE	[31], [143], [144]
*THRB*	OMIM/PubMedGene	OMIM text/BIOCARTA/GENERIF/GENETIC_ASSOCIATION_DB_DISEASE	[145], [146], [147]
*TIMP1*	PubMedGene	GENERIF	[118], [148], [149]
*TIMP3*	PubMedGene	GENERIF	[150], [151], [152]
*TLR2*	OMIM/PubMedGene	OMIM text	[153], [154], [155]
*TLR4*	OMIM/PubMedGene	OMIM text/GENERIF/GENETIC_ASSOCIATION_DB_DISEASE	[155], [156], [157]
*TNFA*	OMIM/PubMedGene	OMIM text	[35], [158], [159]
*TNFB*	OMIM/PubMedGene/PubMedProtein	OMIM text/GENERIF/GENETIC_ASSOCIATION_DB_DISEASE	[160], [161], [162]
*VEGFA*	OMIM/PubMedGene/PubMedProtein	OMIM text/GENETIC_ASSOCIATION_DB_DISEASE	[26], [163], [164]

Consistent with a strong role in the wound healing response, a significant portion of the seed proteins were localized to the ECM. To verify whether our selection of seed proteins was biased, we checked cellular localization of all MI related proteins obtained from OMIM, PubMed Gene, and PubMed Protein databases and encountered a similar result; most of the proteins were localized in the extracellular region and plasma membrane (Table S10). These results indicate that ECM proteins are more likely play a key role in MI response and suggest that our seed protein selection was not biased.

### Construction of MIPIN

From the seed protein list, we searched for all proteins interacting with seed proteins and interactions among the extended proteins through ConsensusPathDB-human, which integrates protein-protein interactions in *Homo sapiens* from different databases such as Intact, DIP, MINT, HPRD, BioGRID and MIPS [22]. Subsequently, we constructed the MIPIN using ‘igraph’ in R [53]. Each vertex of the network represents a protein and each edge between two vertices represents a protein-protein interaction. The resulting MIPIN consists of 613 vertices and 4443 edges.

The degree distribution of MIPIN was examined by the procedure proposed by Clauset et al. and implemented in R [54]. Parameters were estimated based on the theoretical cumulative distribution, 
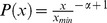
 where *x*, in this case, was degrees of MIPIN vertices. The degree distribution was fitted with *x_min_ = 31* and *α = 3.52* (Kolmogorov-Smirnoff test, *p-value* = 0.97). Additionally, the Kolmogorov-Smirnoff test was performed to examine how well the estimated power law distribution fitted MIPIN vertex degrees. If the Kolmogorov-Smirnoff *p-value*<0.05, we reject the hypothesis that the original data is drawn from the fitted power-law distribution. Otherwise, the higher the Kolmogorov-Smirnoff p-value is above 0.05, the better the estimated power-law distribution fits the data.

### Statistical evaluation

There were several different measures used to characterize the properties of the network, including betweenness centrality, closeness centrality, clustering coefficient, degree centrality, eccentricity, and graph density. The betweenness centrality characterizes the direct and indirect influences of vertices at distant network sites [55]. Closeness centrality measures how many steps are required to access every other vertex from a given vertex [55]. The vertex with the largest value of closeness centrality performs the least amount of steps to sequentially spread information to other reachable vertices from that vertex in the network. Clustering coefficient describes the connectivity of the neighborhood of a vertex [56]. Higher clustering coefficient means more neighbors are connected to each other. Eccentricity of a vertex measured the shortest path distance from the farthest vertex in the graph [57]. We compared the value of six aforementioned measures of MIPIN with the average measurements of randomly generated networks. The empirical *p-values* for each measure were then calculated by counting the number of random networks whose average measures were equal to, greater or smaller than the corresponding values from MIPIN.

### Functional annotation analysis

We examined the functional organization of MIPIN with enriched GO terms using DAVID Functional Annotation Tool [27]. In DAVID, we set the count to be 2 and 0.05 for EASE, a modified Fisher Exact P-Value. We further adapted the method proposed by Louie et al. to measure the specificity of the enriched GO terms for the MIPIN [33]. We computed four measurements to describe the function specificity of enriched GO term lists.

#### i. Number of ancestors

This measurement was calculated by counting the number of ancestor terms for a given GO term up to and including the root term (GO:0008150 : biological processes).

#### ii. Offspring score

Similarly, the number of offspring nodes that a node *t* has was counted as 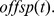 An adjusted measurement of GO offspring was calculated as,
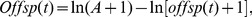
(1)where A was the number of offspring of the root node, which was 23877 for the current version of GO.

#### iii. GO proportion

In order to take into account both the number of ancestor and offspring nodes for a particular GO term *t*, we defined the GO proportion as,
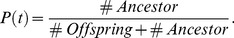
(2)The GO proportion will range from 0 to 1, where 0 indicates non-specific function and 1 indicates high specificity.

#### iv. Information content

The information content (IC) was calculated as follows,
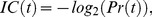
(3)where *Pr(t)* was the ratio of the number of proteins assigned to the term *t* to 14673 human proteins annotated with GO Biological Process. *Pr(t)* was understood as the probability of observing a term *t* in GO dataset. For example, the root term GO:0008150 would have Pr of 1 and IC value of 0. An IC value of NA indicates that the GO term is either obsolete or not available in the current *gene2go* database. A GO term with higher IC value represents more specific function.

### Integration of experimental results and predictions of protein changes

We searched the key word “(myocardial infarction) AND (plasma OR serum)” on PubMed with “Homo Sapiens” as species from Jan 1, 2005 until May 31, 2013. This search resulted in 4326 abstracts. To reduce laborious manual effort, we developed a data mining program written in R using available XML parser and text mining software [58], [59].

The program required two input files, a list of protein aliases and a dictionary of words. We took advantage of a feature offered by UniProt in which users can submit a list of proteins and receive their full names and aliases in XML format. In order to obtain the full names and aliases of MIPIN proteins, we wrote a Java program to parse downloaded UniProt XML files and extract relevant information. The Java program can also be used to retrieve other protein features such as protein structures, domain, and citations in PubMed. The dictionary of words contained commonly used word indicating protein changes such as, “elevate” or “up-regulate” for positive change “UP”, or “down-regulate” or “inhibit” for negative change “DOWN” (Table S7). Abstracts in “txt” format were initially broken into separate sentences. If words of change and names of any proteins were found in the same sentence, we recorded the protein names with the associated words, and PubMed ID of the abstracts. The final output was manually checked to ensure complete and accurate reporting of available protein concentrations. This program significantly reduced the reading time of 4326 abstracts to extracted key sentences.

From these abstracts, we retrieved a small number of proteins with quantified concentrations in plasma or serum post-MI and assigned as labeled proteins in the MIPIN. A large number of MIPIN proteins did not have quantified concentrations and were assigned as unlabeled proteins. We applied semi-supervised learning to predict unlabeled proteins with the labeled protein set. The key component of this method is defined in the similarity matrix. The similarity matrix represents pair-wise similarity or dissimilarity between pairs of vertices. In this case, we combined graph structure similarity matrix evaluated using Jaccard coefficients and functional similarity matrix evaluated using Wang's method [60].

The Jaccard similarity matrix *J* of a graph *G* is a |V(*G*)|×|V(*G*)| square matrix, where |V(G)| denotes the number of vertices in the graph *G*. The Jaccard similarity coefficient of two vertices/proteins *i* and *j* was defined as,
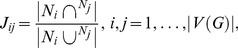
(4)where N_i_ and N_j_ represented the set of direct neighbors of vertex *i* and *j*, respectively [61]. It follows that the diagonal of matrix J is 1.

Besides structural information embedded in the Jaccard similarity matrix, we also integrated biological functions obtained from GO terms by calculating GO biological process similarity matrix *GS* also of size |V(*G*)|×|V(*G*)|.

The pairwise functional similarity between protein *i*, annotated by GO biological process term sets *GOBP_i_* = (*gobp_i1_*, *gobp_i2_*,…,*gobp_im_*), and protein j, annotated by GO biological process term sets *GOBP_j_* = (*gobpj_1_*, *gobpj_2_*,…,*gobp_jn_*), is defined as,
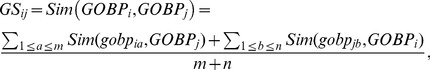
(5)where *Sim(gobp_i_,GOBP_j_)* was defined as the maximum semantic similarity between term *gobp_i_* and any of the terms in set *GOBP_j_*, with *m* and *n* represented terms in the *i* ^th^ and *j* ^th^ GOBP term sets, respectively [60]. The semantic similarity between a pair of GO terms can be determined based on their locations in the directed acyclic GO graph and their semantic relations, which can be ‘is-a’ or ‘part-of’, with their ancestor terms. The GS matrix is symmetric. We chose the Wang method, because the measurement algorithm offered two advantages. First, it only depends on the relationship of the GO terms within a specific ontology, which is the biological process in this case. Second, it avoids the effect of shallow annotation on the semantic relationships between child and parent terms (i.e., with the same parent, a pair of terms near the root should have larger semantic differences than a pair of terms far away from the root). Thus, the algorithm provided a consistent semantic similarity measurement between a pair of GO terms.

We combined Jaccard similarity matrix *J* and GO biological process similarity matrix *GS* to produce the final similarity matrix *W*. The *ij* element of final similarity matrix *W* was defined as,
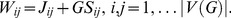
(6)


Let L denote the labeled proteins and U denote the unlabeled proteins. The similarity matrix W could be partitioned as
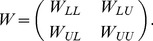
(7)


Let 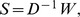 where *D* was the diagonal row sum matrix of *W*, and 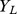 was a binary vector describing the concentrations of labeled proteins post-MI with 1 for positive change “UP” and 0 for negative change “DOWN”. Then the predicted concentration vector 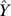 can be computed using the fits algorithm,
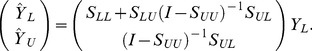
(8)


The predicted concentrations were further updated with the sequential predictions algorithm to drive the estimates towards global point estimates. The algorithm ranked the unlabeled data into *k* number of regions, such that the unlabeled set connecting to the most number of labeled proteins was employed first with the fits algorithm, and penalized unlabeled proteins farther away from labeled proteins with inverse regularization penalty *l*. It was reasonable to initialize the fits algorithm with the protein having the highest labeled connectivity, and repeat with each subsequently ranked protein. We assigned the number of regions *k* to be the number of unlabeled proteins. Since we wanted to maintain a moderate regularization, the inverse regularization penalty *l* was set to be 2. The prediction process was implemented with the package ‘spa’ in R [62].

### Pathways classification

A total of 48 enriched Biocarta pathways were retrieved from DAVID using 613 proteins in MIPIN with ‘Count’ set to be 2 and EASE set to be 0.05. The relationships between proteins and associated pathways could be simplified to a binary matrix of *M* rows and *n* columns, where *M* was the number of enriched pathways and *n* was the total number of associated proteins with enriched pathways (Table 5). If a protein was involved in a pathway, the corresponding score was denoted as 1, otherwise 0. Based on the pathway matrix, we used Kappa statistics to evaluate pathway pairwise similarity matrix based on the belief that pathways sharing common proteins might be related to one another [63].

**Table 5:** An example on evaluation of pathway pairwise similarity matrix using Kappa statistics.

	Protein 1	Protein 2	…	Protein *n*
**Pathway 1**	1	0		1
**Pathway 2**	0	1		0
**…**				
**Pathway ** ***M***	1	1		0

The relationships between proteins and associated pathways were represented as a binary matrix of size *M×n*, corresponding to *M* enriched pathways and *n* associated proteins. The absence and presence of a protein in a pathway were denoted as 0 and 1, respectively.

Considering two pathways *I* and *J* (*I≠* *J* *; I,* *J* *= 1, 2,…, M*), we could determine the number of proteins annotated by both pathways, the number of proteins annotated by pathway *J* but not *I*, the number of proteins annotated by pathway *I* but not *I*, and the number of proteins not annotated by neither pathway among the union of proteins annotated by all pathways, denoted as *a*, *b*, *c* and *d*, respectively. Kappa score κ was defined as 
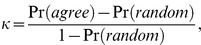
 where Pr(*agree*) was the observed percentage agreement and Pr*(random)* was the overall probability of random agreement. A high Kappa score indicated that two pathways share many common proteins and vice versa.

The observed percentage agreement Pr(*agree*) could be calculated as,
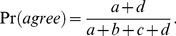
(9)


To calculated the overall probability of random agreement Pr(*random*), we noted that pathway *α* annotates 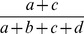 and pathway *β* annotates 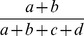 of total associated proteins. Thus, the probability that both pathways randomly annotate the same proteins was 
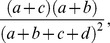
 and the probability that neither pathway randomly annotate the same proteins was 
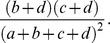
 Thus, the overall probability of random agreement Pr(*random*) could be calculated as,
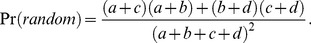
(10)


Kappa score κ could be rewritten as, 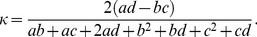
(11)


## Supporting Information

Figure S1 Histograms of log of closeness centrality of vertices in 25 random networks, which resembled delta function. Random networks tend to have outliers.
(EPS)

Figure S2 Plot of closeness centrality against degree. Degree centrality of a vertex in random networks does not have any impacts on its closeness centrality.
(EPS)

Figure S3 MI knowledge map. The MI-specific protein-protein interaction network is depicted with important proteins, enriched biological processes, and cross-talk between different processes. Important proteins are represented as round rectangles with lighter shades. Color codes correspond to different pathways as in Figure 8. For example, VEGFA was involved in both hypoxia and angiogenesis pathways; therefore, it is located in the hypoxia box and colored as green for angiogenesis. The inflammatory response component contains two subgroups, as clustered in Figure 8. The kinase pathways component contains four subgroups. Teal nodes represent proteins which need to be further studied in the future since they are not found enriched in current Biocarta pathways. Details on protein memberships can be found in Table S8.
(EPS)

Table S1 GO cellular component terms of MIPIN seed proteins. We did not combine child terms into their parent terms since they showed more specific functions.
(XLSX)

Table S2 Top 20 enriched GO biological process terms with highest number of ancestors, sorted by the number of ancestors.
(XLSX)

Table S3 List of 80 enriched GO biological process terms with 1 offspring, sorted by *p*-value.
(XLSX)

Table S4 Top 20 GO enriched biological process terms with highest GO proportion.
(XLSX)

Table S5 Top 20 enriched GO biological process terms with highest information content (IC), sorted by IC.
(XLSX)

Table S6 Results from data mining. Expression levels of the top 22 proteins were confirmed with at least two research articles and without any controversial results in PubMed abstracts from the year of 2005 to May 2013. Expression levels of the rest proteins (those listed below the black line) were reported only once or have controversial reports in PubMed abstracts.
(XLSX)

Table S7 Words of changes used for data mining.
(XLSX)

Table S8 MI knowledge map protein membership. Note: While some proteins might be involved in different pathways, we showed only the relevant relationships and not all possible relationships.
(XLSX)

Table S9 (**a**) List of genes obtained from OMIM database with OMIM ID, gene symbol, official name, HUGO ID, and corresponding reviewed Homo sapiens Uniprot ID, (**b**) List of genes obtained from PubMed Gene database with gene symbol, official name, HUGO ID, and corresponding reviewed Homo sapiens Uniprot ID, (**c**) A. List of RefSeq Protein accession obtained from PubMed Protein database, B. List of reviewed Homo sapiens Uniprot ID obtained from RefSeq Protein Accession.
(XLSX)

Table S10 List of enriched Gene Ontology Cellular Component terms in the list of MI-related genes obtained from OMIM, PubMed Gene and Protein databases.
(XLSX)
